# Diabetes-Induced Superoxide Anion and Breakdown of the Blood-Retinal Barrier: Role of the VEGF/uPAR Pathway

**DOI:** 10.1371/journal.pone.0071868

**Published:** 2013-08-07

**Authors:** Azza B. El-Remessy, Telina Franklin, Nagla Ghaley, Jinling Yang, Michael W. Brands, Ruth B. Caldwell, Mohamed Ali Behzadian

**Affiliations:** 1 Vascular Biology Center, Medical College of Georgia, Georgia Regents University, Augusta, Georgia, United States of America; 2 Culver Vision Discovery Institute, Medical College of Georgia, Georgia Regents University, Augusta, Georgia, United States of America; 3 Department of Cellular Biology & Anatomy, Medical College of Georgia, Georgia Regents University, Augusta, Georgia, United States of America; 4 Department of Physiology, Medical College of Georgia, Georgia Regents University, Augusta, Georgia, United States of America; 5 Clinical and Experimental Therapeutics, University of Georgia, Augusta, Georgia, United States of America; 6 Charlie Norwood VA Medical Center, Augusta, Georgia, United States of America; Cedars-Sinai Medical Center, United States of America

## Abstract

Diabetes-induced breakdown of the blood-retinal barrier (BRB) has been linked to hyperglycemia-induced expression of vascular endothelial growth factor (VEGF) and is likely mediated by an increase in oxidative stress. We have shown that VEGF increases permeability of retinal endothelial cells (REC) by inducing expression of urokinase plasminogen activator receptor (uPAR). The purpose of this study was to define the role of superoxide anion in VEGF/uPAR expression and BRB breakdown in diabetes. Studies were performed in streptozotocin diabetic rats and mice and high glucose (HG) treated REC. The superoxide dismutase (SOD) mimetic tempol blocked diabetes-induced permeability and uPAR expression in rats and the cell permeable SOD inhibited HG-induced expression of uPAR and VEGF in REC. Inhibiting VEGFR blocked HG-induced expression of VEGF and uPAR and GSK-3β phosphorylation in REC. HG caused β-catenin translocation from the plasma membrane into the cytosol and nucleus. Treatment with HG-conditioned media increased REC paracellular permeability that was blocked by anti-uPA or anti-uPAR antibodies. Moreover, deletion of uPAR blocked diabetes-induced BRB breakdown and activation of MMP-9 in mice. Together, these data indicate that diabetes-induced oxidative stress triggers BRB breakdown by a mechanism involving uPAR expression through VEGF-induced activation of the GSK3β/β-catenin signaling pathway.

## Introduction

Breakdown of the blood-retinal barrier (BRB) occurs early in diabetic retinopathy and leads to vascular leakage and retinal edema [1,2]. The vascular permeability defect has been attributed to elevated blood glucose levels (for review, please see 3). Increases in vascular endothelial growth factor (VEGF) are also evident in retinal tissue and ocular fluids of diabetic patients and animals and are likely mediated by an increase in oxidative stress [4–9]. Although diabetes- and high glucose-induced increases in superoxide anion have been well-documented [10–12], the specific relationship between superoxide anion generation and BRB breakdown has not been elucidated. Anti-VEGF therapies have shown promise in reducing vascular leakage and macular edema in diabetic patients. However, in light of the potential for adverse effects with repeated anti-VEGF injections and the beneficial actions of VEGF as a survival factor (reviewed in 13), there is great need for additional anti-permeability therapies. Thus, a more precise definition of the mechanisms involved in the diabetes-induced permeability increase is needed.

We and others have shown that diabetes-induced retinal vascular permeability and VEGF-induced paracellular permeability in retinal endothelial cells are accompanied by increases in expression of the receptor for urokinase plasminogen activator (uPAR) [4,14–17]. Urokinase (uPA) is a serine proteinase that is expressed constitutively in endothelial cells. It is secreted in latent pro-form as a single chain 50kDa peptide, but is rapidly activated upon binding to uPAR. Upon activation, uPA converts plasminogen to plasmin. Plasmin activates several pro-forms of matrix metalloproteinases (MMPs), generating a cascade of proteinase activation at the cell surface [18,19]. This leads to degradation of the extracellular matrix and disruption of cell-cell and cell-matrix attachments. The role of this proteolytic cascade in diabetes-induced breakdown of the blood-retinal barrier has been supported by studies showing that treatment with inhibitors of MMP or uPA blocks diabetes-induced breakdown of the BRB [15,20], but the upstream mediators of this process are as yet unknown.

Vascular endothelial cell paracellular permeability function is regulated by adherens and tight junctions [21]. In adherens junctions, β-catenin links the intracellular domain of VE cadherin to actin microfilaments via α-catenin [22]. In addition to this structural role, β-catenin acts as an intracellular signaling molecule and is involved in regulating cell proliferation and differentiation. In differentiated cells, β-catenin is predominantly bound to the plasma membrane and free cytosolic β-catenin is phosphorylated by GSK3β (glycogen synthase kinase) which targets it for ubiquitination and proteosomal degradation [23,24]. Upon growth factor or Wnt signaling, GSK3β is phosphorylated and deactivated. Under these conditions, β-catenin escapes ubiquitination, accumulates in the cytosol and translocates into the nucleus where it serves as a co-transcription factor to activate a variety of genes associated with cell migration and proliferation, including uPAR [25]. Based on these observations, we hypothesized that diabetes/high glucose increases superoxide anion formation that drives VEGF expression and retinal vascular permeability by activating the GSK3β, β-catenin, uPAR pathway. We tested this hypothesis by studies with inhibiting superoxide anion in vivo and in high glucose-treated endothelial cells as well as uPAR knockout diabetic mice.

## Materials and Methods

### Cell Culture

Primary cultures of bovine retinal microvascular endothelial cells (REC) were prepared according to our established protocol [14,26]. Prior to all experimental procedures, medium was switched to a serum-free endothelial basal medium (EBM, Clonetics, San Diego CA) including 0.1% BSA, with or without 25 mM glucose and various inhibitors for the designated times. The high glucose treated REC-conditioned media (HGCM) were prepared by incubating the cells with high glucose (25 mM) for 2-3 days. Control conditioned media were prepared by incubating cells for the same length of time in serum free (EBM/BSA) medium with normal glucose (5.5 mM glucose) or medium containing L-glucose (19.5 mM L-glucose, 5.5mM D-glucose; final concentration of 25 mM). Conditioned media were collected and concentrated 10 fold by spin-filtration (10 kDa cutoff membrane, Millipore UFV4BK10) and added to the cultures. The cell permeable superoxide dismutase (PEG-SOD) was purchased from Sigma was used at 80U/ml. VEGFR1/2 tyrosine kinase inhibitor SU5416 (Tocris) was used at 2 µM.

### Quantitative RT-PCR

Quantitative mRNA analysis by real-time PCR was carried out as described previously [9,14,16,17]. Briefly, total RNA was extracted and stored in ethanol. Aliquots of 2 µg total RNA were treated with DNase and reversed transcribed by using random primers. A pair of primers flanking 250 nucleotides on the bovine uPAR cDNA sequence (nt. # 270-520, NCBI- AF144762) was designed. The amplicon generated by conventional PCR was cloned and was used as standard template in quantitative PCR. Similarly, VEGF primers were designed based on a bovine cDNA sequence (nt. # 230-430, NCBI-M31836) and the amplicon generated by conventional PCR was purified and used as standard. For rat uPAR expression, primers were (5'-GGA CCA ATG AATCAG TGC TTG-3', 5'-CCA CAG TCT GAG GGT CAGGAG-3', length of product is 252 bp). For mouse MMP-9 expression, primers were 5′-AAATGTGGGTGTACACAGGC-3′ and 5′-TTCACCCGGTTGTGGAAACT-3′. Quantitative PCR was carried out in a Light Cycler apparatus (Roche Diagnostics, Indianapolois, IN) using a kit provided by the same vendor, or in Smart Cycler (Cephied, Sunnyvale, CA); each reverse transcription (RT) preparation was used twice and in duplicate. The results were normalized with corresponding internal markers and averaged.

### Western Blot

The presence of VEGF in conditioned media was examined by Western blotting. Concentrated samples were adjusted for protein content and loaded on 12% acrylamide gels. Membranes were incubated with anti-VEGF antibody (Santa Cruz Biotechnology, Dallas, TX) and developed by enhanced Chemiluminescence (ECL). Two major protein bands corresponding to VEGF monomer (~ 21 kDa) and dimer (~ 42 kDa) were identified and were scanned for densitometry.

For analysis of GSK3β, RECs grown in 100 mm diameter dishes were treated as designated in the results section. At the end of treatment, cells were collected and homogenized in a lysis buffer consisting of 50 mM Tris-HCl, pH:7.4, 150 mM NaCl, 0.25% deoxycholic acid, 1% NP-40, 1 mM EDTA, 1 mM PMSF, 1 mM sodium orthovanadate, 1 mM sodium orthophosphate, to which protease inhibitor cocktail was added. The homogenates were centrifuged at 15,000 xg and the supernatants were collected. Aliquots were taken for protein assay and the remaining material was used immediately or stored at -70° C. Anti-GSK3β antibody (Santa Cruz Biotechnology) and protein-A/G conjugated sepharose beads were added to the cell extracts and placed in a shaker at 4^o^C overnight. After washing, the beads were directly boiled in SDS sample buffer and applied to SDS PAGE followed by electro-blotting and immunoblotting for phospho-GSK-3β. Membranes were stripped and reprobed for GSK-3β to check for equal loading.

### Analysis of β-catenin Subcellular Distribution

Endothelial cells were grown in gelatin coated chamber slides in the absence or presence of high glucose for 5 days. They were fixed and processed for immunocytochemistry using anti β-catenin antibody and fluorescein-conjugated secondary antibody as described before [14,17]. Subcellular distribution of β-catenin was analyzed by using a MultiProbe 2001 confocal laser scanning microscope (Molecular Dynamics, Sunnyvale CA). Isolation of nuclei, extraction and Western blot analysis of nuclear β-catenin has been described previously [14,17].

### Trans-endothelial Electrical Resistance (TER)

Cell permeability was assessed by measuring changes TER using ECIS (electrical cell-substrate impedance sensing, Applied Biophysics, Troy, NY). Cells were grown in 8-well chamber slides equipped with gold-coated micro-electrodes. The electric current passing through the endothelial monolayers was measured independently in each chamber. TER was measured continuously and in real time before, during and after the treatment of the cells. When neutralizing antibodies were used (anti-uPA # SC-6830 and anti-uPAR # SC-9793, Santa Cruz Biotechnology; 4 µg/ml), they were added 30 min before the treatments.

### Gelatin Zymography

Media conditioned by RECs and vitrectomy samples from mice were processed for zymographic analysis of MMP activity as described previously [26]. Briefly. SDS-polyacrylamide gels were copolymerized with 0.1% gelatin, and electrophoresis of samples equated for protein was carried out under non-reducing conditions. Gels were rinsed in 50 mM Tris buffer (pH 7.5) containing 2.5% Triton X-100 to remove SDS, incubated in reaction buffer at 37° C overnight and stained with brilliant blue G250. Enzyme activities were revealed as clear bands (lysis zone) against the dark blue background of the substrate gel. For control, duplicate samples were applied to gelatin gels. After electrophoresis, gels were cut into halves and were developed in the presence and absence of EDTA.

### Animals

This study was carried out in strict accordance with the recommendations in the Guide for the Care and Use of Laboratory Animals of the National Institutes of Health. The Animal Protocol #2008-245 was approved by the Institutional Animal Care and Use Committee of Georgia Regents University (Animal Welfare Assurance no. A3307-01). All surgery was performed under avertin anesthesia, and all efforts were made to minimize suffering.

### Rat Studies

Male rats (weight, 325 to 350 g, Harlan Sprague-Dawley) were catheterized to receive continuous infusion as described previously [27]. Briefly, anesthesia was induced with sodium pentobarbital (50 mg/kg IP) and atropine (40 µg IP per rat) was administered to ensure an unobstructed airway. Under aseptic conditions, artery and vein catheters were implanted. Animals were randomized into three groups: control (n=6), diabetic (n=8) and diabetic treated with the SOD mimetic 4-hydroxy-tempo (tempol, Sigma-Aldrich, St Louis, MO, n=6). Diabetes was induced by streptozotocin (40 mg/kg IV). After the first day of diabetes, tempol (18 µmol/kg per hour IV) was added to the infusate of one of the diabetic groups. Control and diabetic groups received sterile 0.9% saline. Animals were sacrificed after 2-weeks. Treatment with tempol did not alter blood glucose levels. Average blood glucose in the saline-treated diabetic group was 441±46 mg/dL compared with 501±10 mg/dL in the diabetic+tempol group and 114±4 mg/dL in the control group.

### Mouse Studies

Experiments were performed with uPAR-deficient mice on a C57Bl/6 background (Jackson Laboratories, Bar Harbor, ME) and their age-matched congenic controls. Genotyping was done prior to experiments. Age-matched uPAR^-/-^ and control mice (25g) were made diabetic by intraperitoneal injections of 55mg/kg streptozotocin (STZ, Sigma) dissolved in 0.1M fresh citrate buffer, pH 4.5. Animals were considered diabetic when their plasma glucose level exceeded 250 mg/dL. Animals were studied after 5-8 weeks of hyperglycemia. The uPAR gene deletion had no effect on body weight or blood glucose level in diabetic rats. The uPAR and congenic control diabetic animals had significant increases in blood glucose levels (487+34 and 502+39 mg/dL, respectively) compared with the uPAR and congenic control non-diabetic mice (185+8 and 189+12 mg/dL, respectively). Weights of the diabetic uPAR-/- and congenic control mice at the end of the experiments were 22+2 and 20+2 grams vs 28+1 and 26+1 in the normoglycemic uPAR-/- and congenic control mice.

### Permeability Assay

Diabetic and age-matched normoglycemic control rats or mice were processed for quantification of permeability as described previously [4,28]. The mice were anesthetized with avertin (2.5%) administered intraperitoneally at 0.015 ml/g body wt. The rats were anesthetized with Ketamine/Xylazine administrated intraperitoneally. Animals were then injected intravenously with 10 mg/kg bovine serum albumin (BSA)-Alexa-Fluor 488 conjugate (Molecular Probes, Eugene, OR). After 30 minutes, animals were sacrificed and the eyes were removed, embedded in OCT embedding medium, snap frozen in liquid nitrogen. Frozen sections (12 µm) were prepared. Images were collected at 60 µm intervals from 10 sections of each retina. Tracer extravasation was measured by morphometric analysis of fluorescein levels in non-vascular areas in each image. Fluorescein intensity in the retinal sections was normalized according to the concentration of the tracer in the plasma of the same animal.

### Oxidative Stress Markers

Levels of lipid peroxides (malondialdehyde, MDA) were assayed using thiobarbituric acid reactive substances as described before [10,29]. Briefly, plasma or retinal homogenate were acidified with 20% acetic acid, 8% sodium dodecyl sulfate, and thiobarbituric acid at 95° C for 60 min. The samples were centrifuged, the supernatant was extracted with n-butanol and pyridine (15:1, respectively), and the absorbance of the organic solvent layer was measured colorimetrically at 532 nm. The results were compared to an external standard (tetramethoxypropane). The Bradford assay (Bio-Rad, Hercules, CA) was also performed to determine the protein concentration of the retinal lysate. The lipid peroxide level was expressed in µM MDA/mg total protein. Slot-blot analysis was used to detect retinal nitrotyrosine formation as described previously [4], 5µg of retinal homogenate were immobilized onto a nitrocellulose membrane. After blocking, the membrane was reacted with anti-nitrotyrosine polyclonal antibody (Calbiochem, Millipore) followed by its secondary antibody, and the optical densities of samples were compared to those of the controls.

### Statistical Analysis

The results are expressed as mean ± SEM. For statistical analysis of tissue culture data, duplicate dishes of cells were treated separately in each experiment and all experiments were repeated at least once or twice to generate 4 to 6 independent data. Differences among groups were evaluated by ANOVA and the significance of differences between groups was assessed by the post-hoc test (Tukey) when indicated. Significance was defined as P < 0.05.

## Results

### Tempol Blocks Diabetes-induced BRB Breakdown

**Figure 1:**
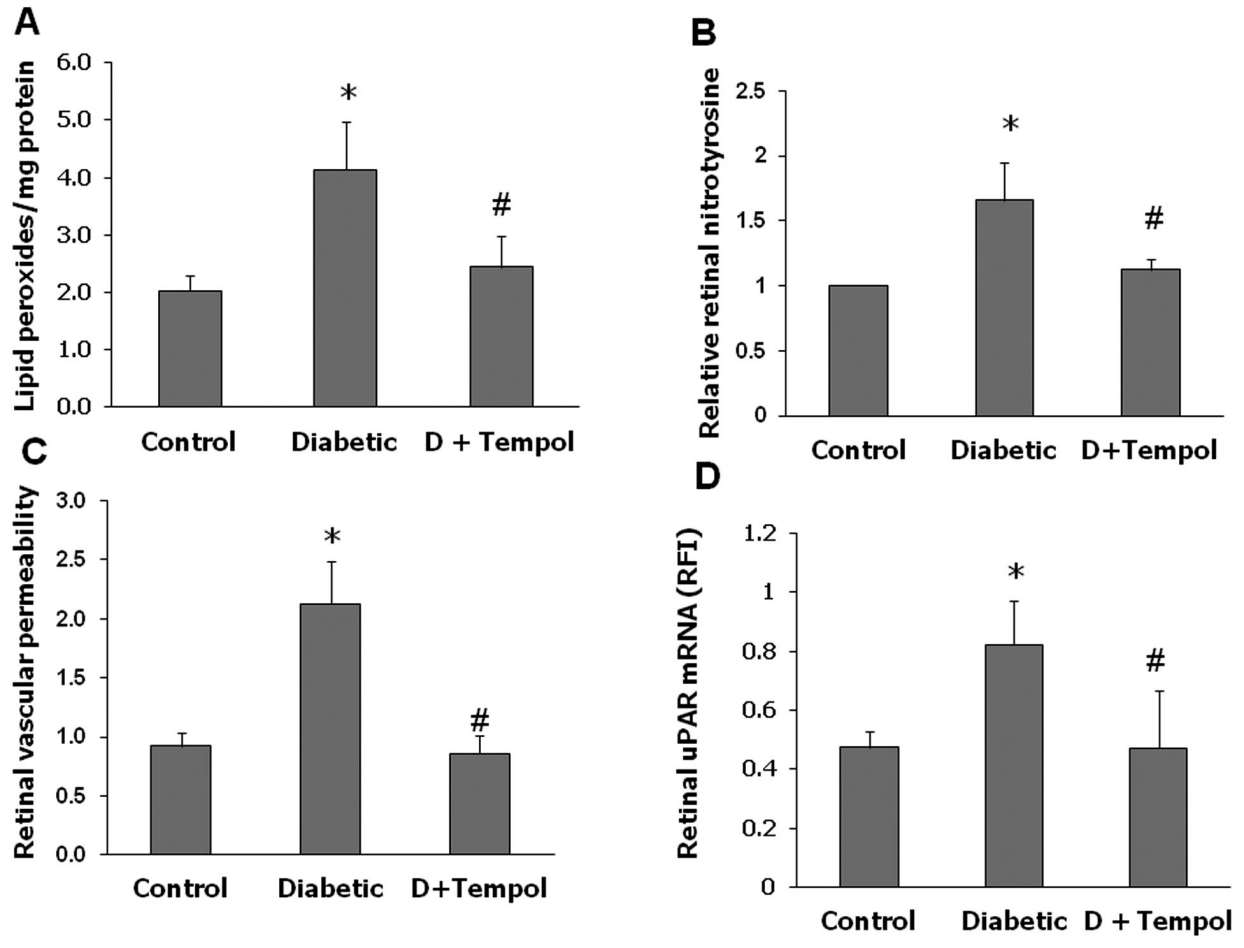
Tempol prevents diabetes-induced oxidative stress and BRB breakdown in rats. Rats were rendered diabetic using STZ and 1 day after being diabetic, continuous infusion of tempol (18 micromoles/Kg/day IV) or saline was started for 2-weeks. A. Lipid peroxidation assessed by TBARS assay showed 2-fold increase in diabetic rat retina compared to controls (n=5-6, P<0.5). Treatment of diabetic animals with tempol blocked these effects. B. Slot blot analysis showed 1.7-fold increase in tyrosine nitration in diabetic rat retinas compared to controls. This effect was blocked by tempol treatment (n=5-6, P<.05). C. Diabetic and non-diabetic rats were injected intravenously with Alexa-Fluor 488-BSA (10 mg/kg) and permeability was quantified as described in the methods section. In comparison to control, permeability was significantly increased 2-fold (n=5-6, P<0.05) in the diabetic group. Treatment with tempol prevented diabetes-induced BRB breakdown. D. Quantitative RT-PCR analysis was performed with RNA isolated from various groups. Diabetes caused ~2-fold increase in uPAR expression that was significantly inhibited by tempol (*P<0.05, n=6, P<0.05).

We have shown previously that diabetes-induced oxidative stress is positively correlated with BRB breakdown [4]. Here we tested the direct effect of inhibiting superoxide anion on vascular permeability by treating diabetic rats with the SOD mimetic tempol. The antioxidant action of tempol was verified by examining lipid peroxidation and nitrotyrosine formation in the retinas from various groups. The diabetic retinas showed a 2-fold increase in lipid peroxidation and a 1.7-fold increase in nitrotyrosine compared to controls (Figure 1A-B). Treatment with tempol completely normalized these indicators of oxidative stress. As shown in Figure 1C, diabetes-induced a significant increase in retina vascular permeability as demonstrated by extravasation of BSA-fluorescein. Treatment of diabetic rats with tempol blocked this effect. Based on our previous studies linking diabetes-induced BRB breakdown to activation of the uPA/uPAR pathway [4], we next examined the effects of inhibiting superoxide anion on expression of uPAR. As shown in Figure 1D, diabetic retinas had a 2-fold increase in uPAR expression compared to controls. This effect was not seen in the tempol-treated diabetic rats.

### SOD Blocks High Glucose-induced VEGF Expression

**Figure 2:**
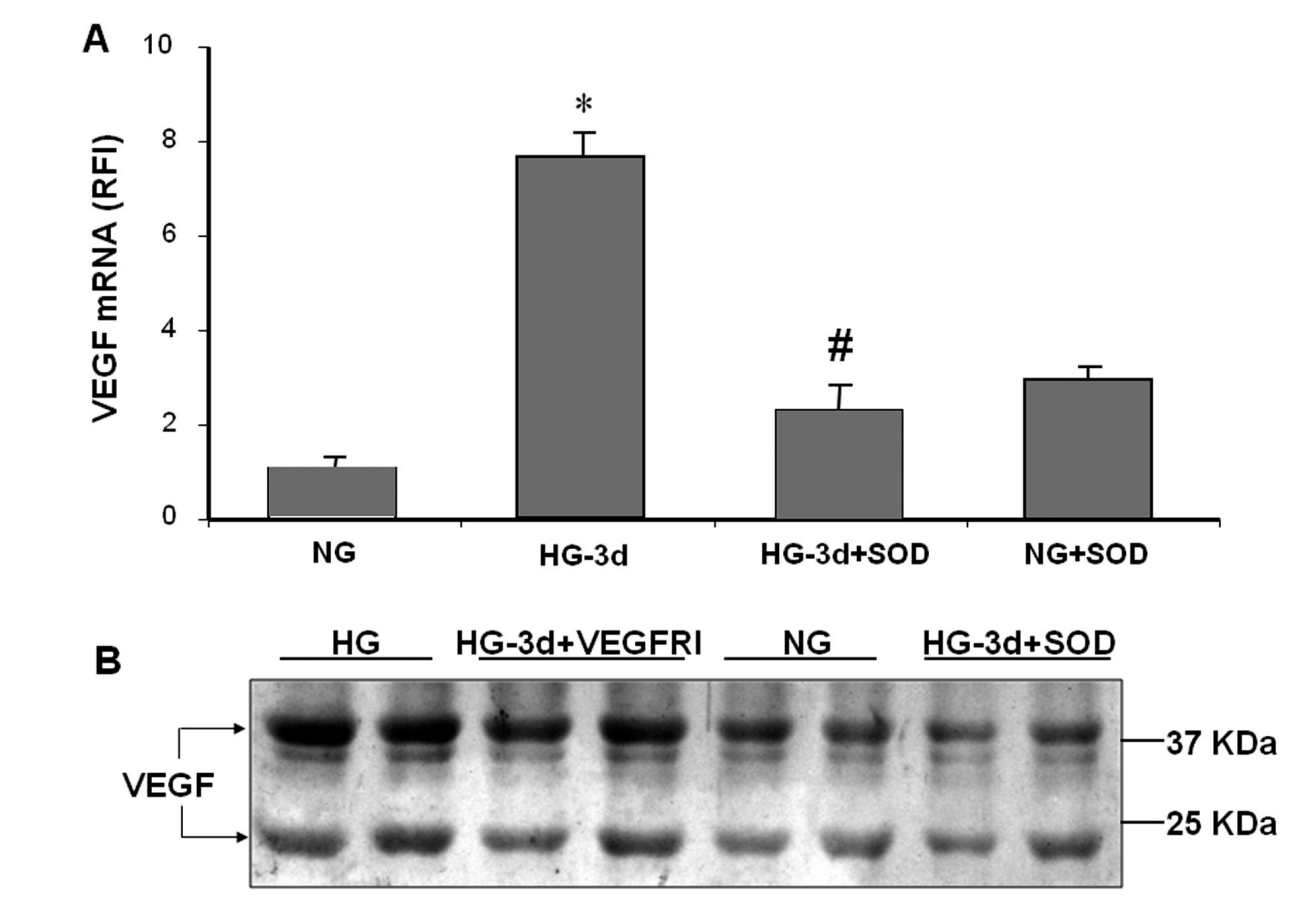
PEG SOD prevents HG glucose induced VEGF expression. Quantitative RT-PCR and Western blotting analyses were performed with RNA and protein isolated from endothelial cells cultured for 3 days in serum-free medium containing 5.5 mM glucose (NG) or 25 mM glucose (HG) with or without superoxide dismutase (SOD, 80 U/ml) or VEGFR inhibitor (VEGFRI, 2 µM). HG treatment increased VEGF mRNA levels as compared with NG treated cells and this effect was blocked by SOD (A). Western blot analysis confirmed increased levels of VEGF in conditioned media from HG treated endothelial cells as compared with the NG control and HG + SOD cultures (B).

To explore the mechanisms by which reducing superoxide anion can preserve BRB function, we used cultures of retinal endothelial cells (REC) maintained in high glucose (HG, 25 mM) or normal glucose (5 mM) for 3-5 days. In response to HG, superoxide anion generation increased and reached a peak on day 3 and remained high through day 5 (data not shown). Superoxide anion generation was inhibited by the cell permeable PEG-SOD (80 U/ml). As shown in Figure 2, high glucose treatment significantly increased VEGF mRNA expression in REC and increased the release of VEGF into the conditioned medium. HG-induced increases in VEGF mRNA and protein were inhibited by SOD, indicating involvement of superoxide formation (Figure 2A). As shown in Figure 2B, the HG-induced upregulation of VEGF was partially blocked by the VEGFR1/2 inhibitor, suggesting that under this experimental condition, part of the increase in VEGF expression involves an autocrine mechanism.

### SOD Blocks High Glucose-induced uPAR Expression

**Figure 3:**
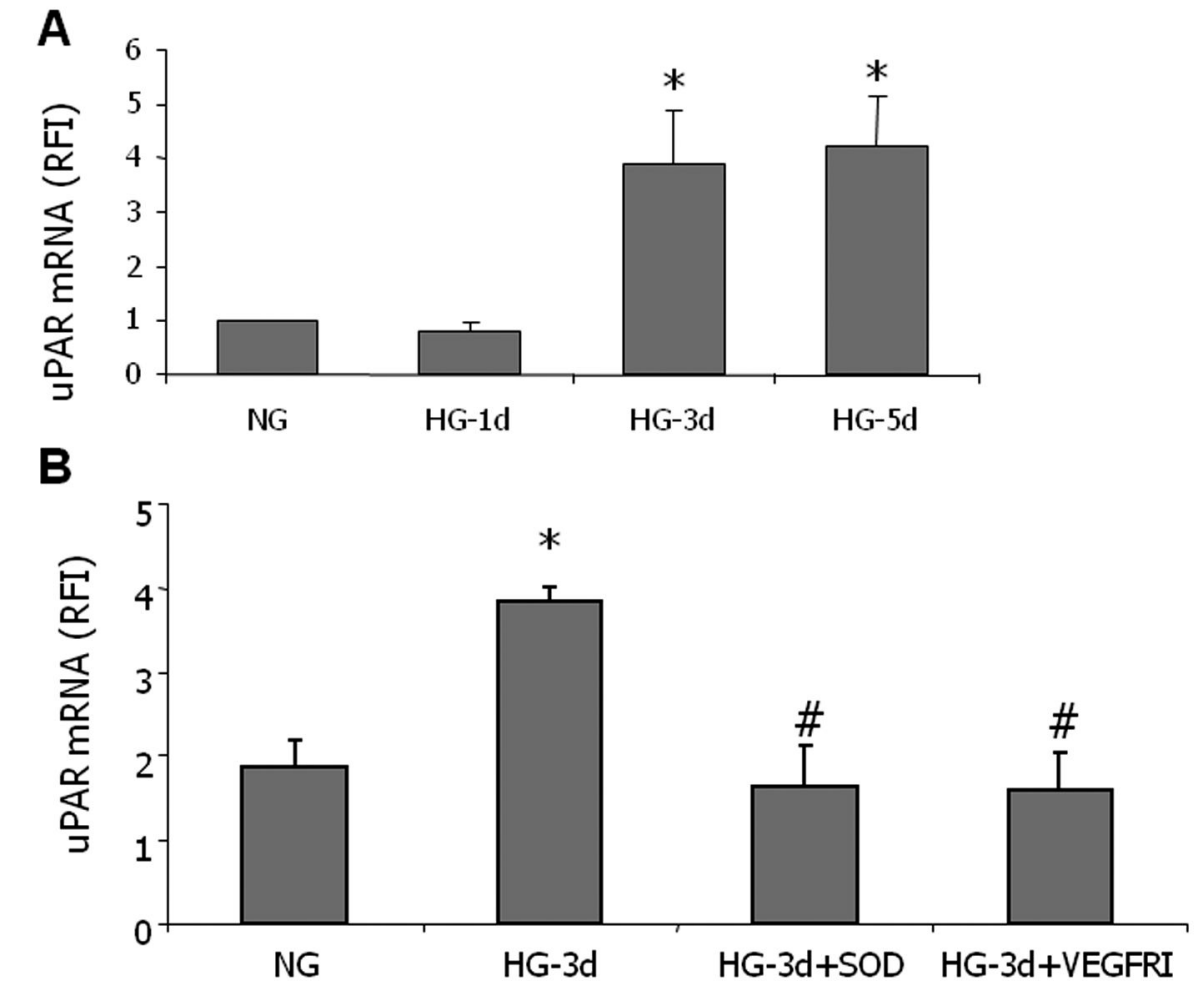
PEG SOD prevents HG induced uPAR expression. Quantitative RT-PCR analysis was performed with RNA isolated from endothelial cells cultured for 1 to 5 days in serum-free medium containing 5.5 mM glucose (NG), 25 mM glucose (HG) with or without superoxide dismutase (SOD, 80 U/ml) or VEGFR inhibitor (VEGFRI, 2 µM). HG treatment caused a time-dependent increase in uPAR (A) that was blocked by SOD or VEGFRI (B).

Based on our previous studies linking VEGF-induced increases in REC permeability to activation of the uPA/uPAR signaling pathway [14,17], we next examined the effects of HG on uPAR expression in REC. As shown in Figure 3A, high glucose treatment induced an increase in uPAR expression which reached a peak on day 3 and remained high through day 5. The high glucose-induced uPAR expression was blocked by SOD or by an inhibitor of VEGFR1/2 tyrosine kinase activity (Figure 3B), suggesting that formation of superoxide anion in glucose treated cells induces uPAR expression and that VEGF expression and receptor activation play a role in this process. Osmotic control experiments showed that treatment with high L-glucose did not alter uPAR expression (data not shown).

### Inhibiting VEGFR Prevents HG-induced Phosphorylation/inhibition of GSK3β

**Figure 4:**
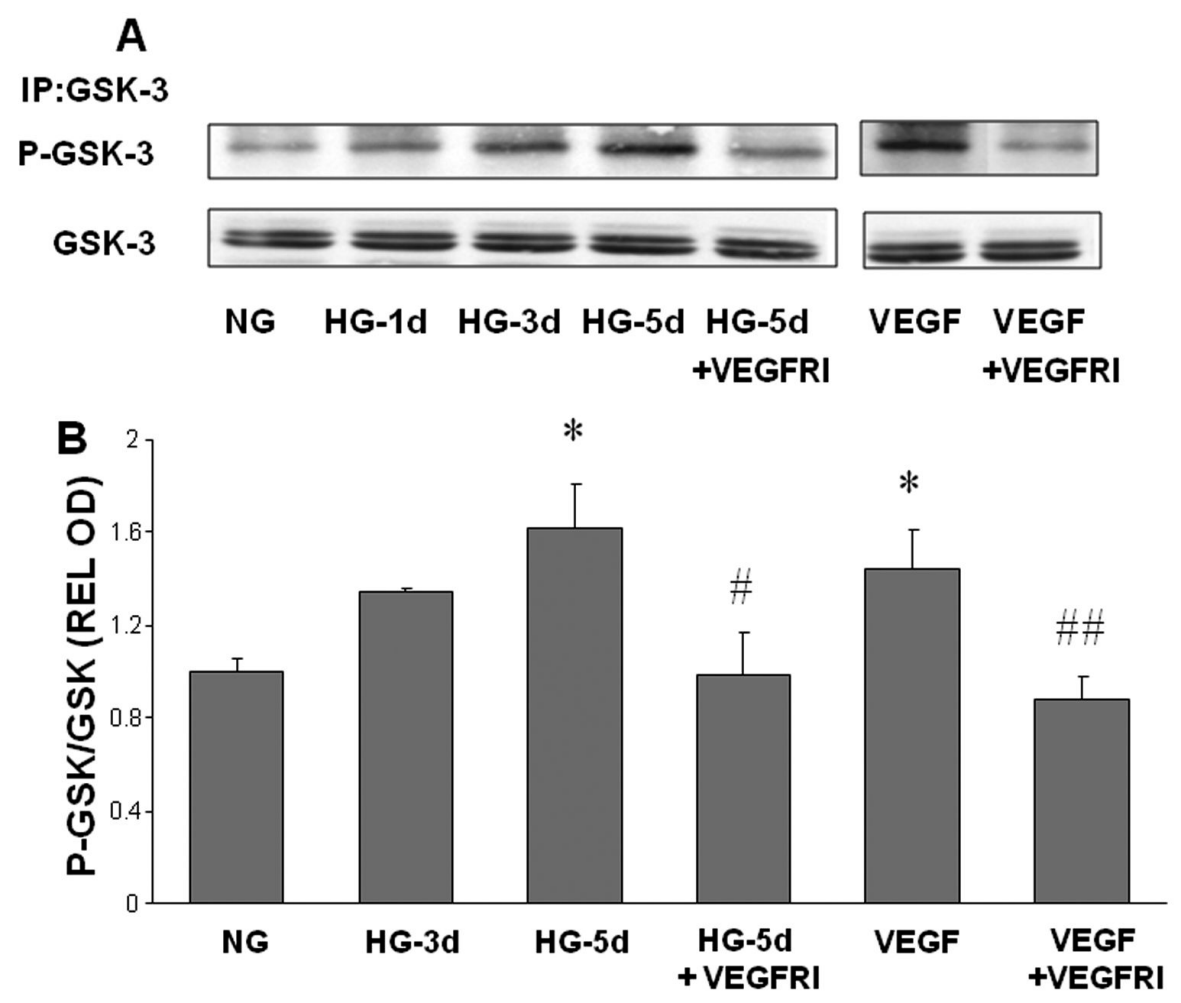
High glucose-induced phosphorylation of GSK3β. Endothelial cells were grown in serum-free medium containing normal glucose (NG, 5.5 mM) or high glucose (HG, 25 mM) with or without VEGFR inhibitor (VEGFRI) for 1 to 5 days. Equal amounts of SDS extracted protein samples were immunoprecipitated (IP) by anti-GSK3β antibody and subjected to SDS-PAGE and Western blotting (A). Densitometric analysis of phospho-GSK3β bands, normalized for the corresponding GSK3β bands, showed that phosho-GSK3β levels were significantly increased by HG or VEGF treatment and that this effect was reduced in VEGFI treated samples compared to HG or VEGF treated endothelial cells (B). * = P<0.05 vs NG, # = P<0.05 vs HG5, # # = P<0.05 vs VEGF.

Under normal physiological conditions, GSK3β targets free cytosolic β-catenin for degradation in proteosomes. Phosphorylation of GSK3β inhibits this function and allows β-catenin to accumulate in the cytosol and translocate into the nucleus to activate gene transcription. As shown in Figure 4, GSK3β phosphorylation was increased in cells treated with HG or VEGF. In these experiments, GSK3β phosphorylation increased gradually over 3-5 days of glucose treatment but was elevated within a few hours after VEGF treatment. The VEGFR1/2 inhibitor blocked GSK3β phosphorylation under both high glucose and VEGF conditions. This observation suggests that VEGF/VEGFR mediates the high glucose-induced phosphorylation/inhibition of GSK3β.

### HG Induces Nuclear Translocation of β-catenin

**Figure 5:**
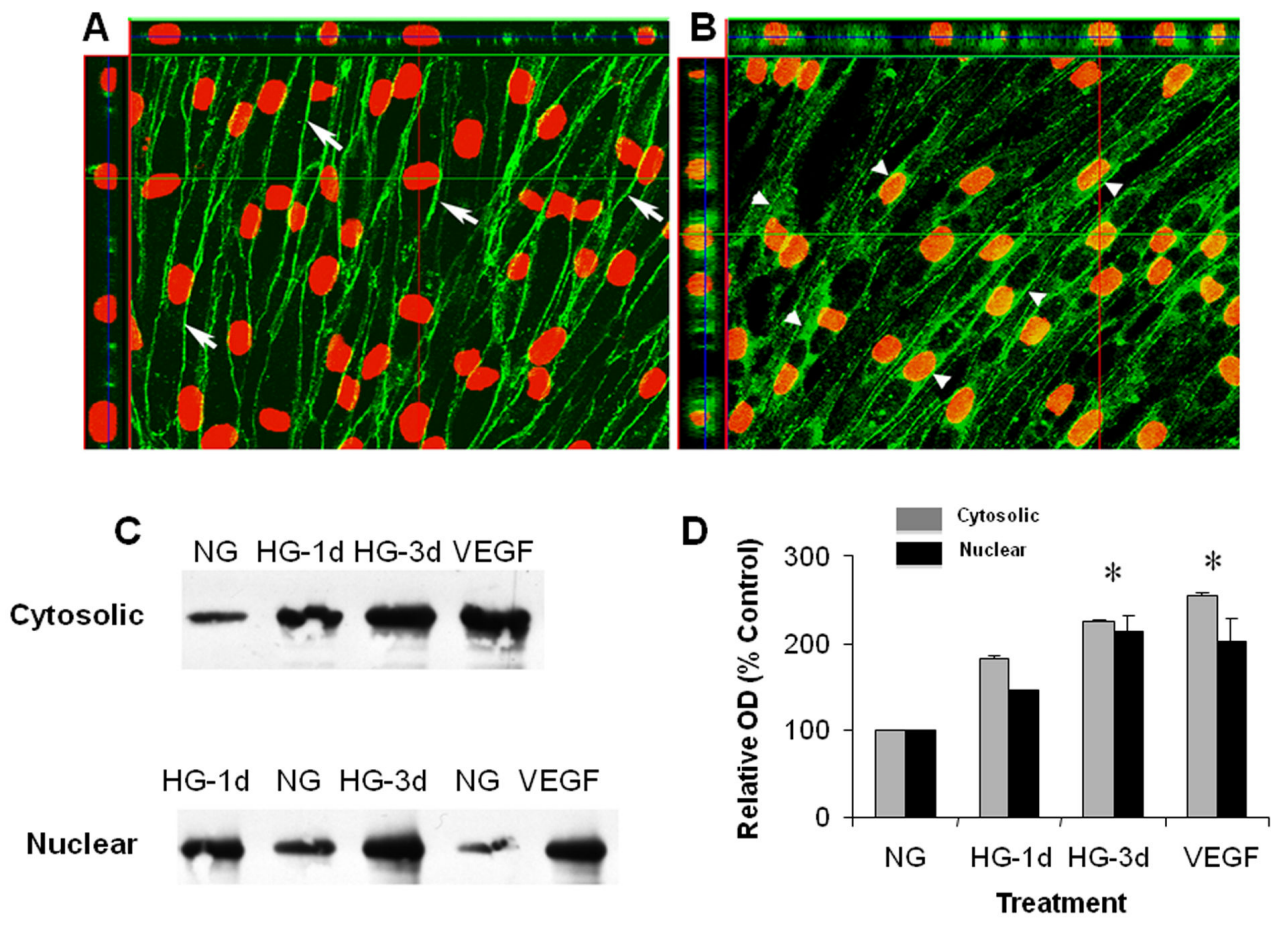
High glucose-induced nuclear translocation of β-catenin. Endothelial cells were grown in serum-free medium with normal glucose (NG, 5.5mM) or high glucose (HG, 25 mM) for 3 days, fixed and processed for immunocytochemistry and confocal microscopy or used for cell fractionation. Confocal imaging of NG cultures using anti β-catenin antibody (green) and propidium iodide nuclear staining (red) shows membrane bound β-catenin (arrows) (A). Following high glucose treatment β-catenin was redistributed into the cytosol (arrowheads) and nucleus (as shown in z-series optical slices above and at left) (B). Western blotting showed increased cytoplasmic and nuclear β-catenin following HG treatment (C). Densitomitric analysis showed significant increases in cytosolic and nuclear β-catenin levels following HG or VEGF treatment (D) (* = P < 0.05 vs Control). Gray bars represent cytosolic β-catenin. Black bars represent nuclear β-catenin.

Our previous studies have shown that VEGF-induced increases in uPAR expression and paracellular permeability are mediated by transcriptional activation of the β-catenin as evidenced by its cytosolic redistribution and nuclear translocation [14,17]. We therefore determined high glucose effects on the subcellular distribution of β-catenin. Confocal imaging analysis showed that β-catenin was localized mainly at the cell-to-cell junctions in endothelial cells maintained in normal glucose medium (Figure 5A). However, the cells maintained in medium with high glucose (3 days) showed conspicuous accumulation of β-catenin within the cytoplasm (Figure 5B). Three-dimensional image analysis also showed the presence of β-catenin within the nuclei. This microscopic observation was confirmed by Western blot analysis showing β-catenin accumulation in cytosolic and nuclear fractions prepared from high glucose-treated cells (Figure 5C, D). These data indicate that high glucose-induced uPAR expression in retinal endothelial cells involves β-catenin transcriptional activity.

### Inhibiting uPA or uPAR Prevents HG-induced REC Permeability

**Figure 6:**
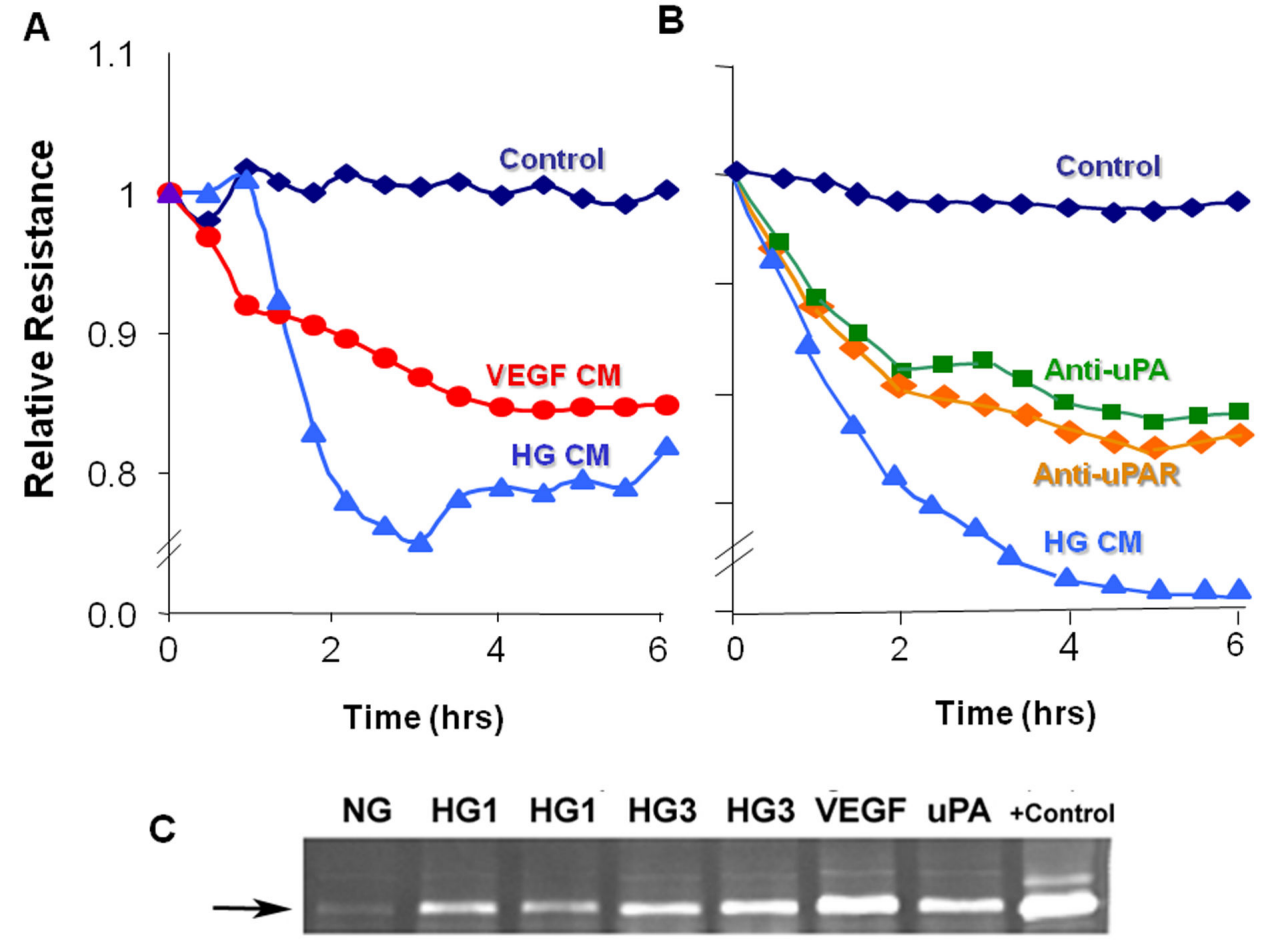
High glucose-induced permeability increase. Endothelial monolayers grown in ECIS microarray slides were treated with conditioned media from endothelial cells treated with high glucose (HG CM) or VEGF (VEGF CM) and tested for effects on TER (transcellular electrical resistance). Serum free medium was used as control. TER was markedly reduced by HG CM or VEGF CM (A). HG CM-induced decreases in TER were prominently reduced by pretreatment of the cultures with anti-uPA or anti-uPAR antibodies (B). Gelatin zymograms prepared using medium conditioned by cells treated with high glucose for 1 day (HG1) or 3 days (HG3) or with VEGF or uPA showed prominent increases in MMP9 activity as compared with medium from normal glucose controls (NG) (C). +Control = MMP9.

In order to assess the direct role of uPAR in REC permeability, we used electrical cell impedance sensing (ECIS) to measure transcellular electrical resistance (TER). This analysis showed that media conditioned by REC cells treated with either HG or VEGF significantly reduced TER levels of the endothelial monolayers, indicating an increase in paracellular permeability (Figure 6A). These results were confirmed by measurement of TER using transwell filter chambers and chopstick electrodes (data not shown). The high glucose effect was inhibited when the target cells were pre-incubated with antibodies against uPA or uPAR (Figure 6B), supporting involvement of the uPA/uPAR pathway in the barrier dysfunction. The blocking effect was partial, perhaps due to inefficacy of the antibodies or the presence of other permeability factors in the conditioned media. The involvement of the uPA/uPAR pathway in the hyperpermeability effect was further supported by zymographic analysis showing increased levels of MMP9 activity in media from the cultures treated with high glucose, VEGF or uPA (Figure 6C). Medium conditioned by control cultures maintained in normal glucose conditions was negative for MMP9. Upregulation of uPAR allows uPA binding and activation, leading to activation of plasmin and MMPs, which are known to increase retinal vascular permeability [26,30].

### Requirement of uPAR Expression for Diabetes-induced Breakdown of the BRB

**Figure 7:**
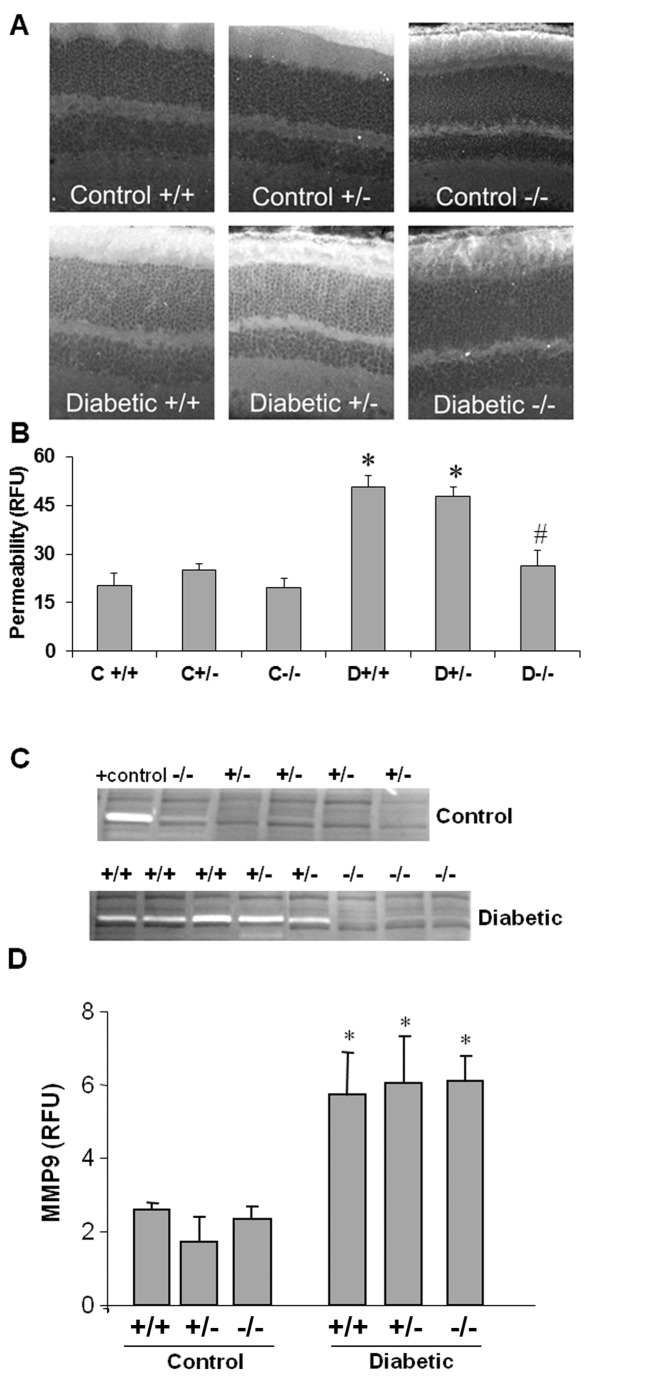
Blockade of diabetes-induced increase in retinal vascular permeability and MMP9 activity in uPAR-/- mice. Diabetic and non-diabetic uPAR-/- mice and their congenic controls (uPAR+/+, uPAR+/-) were injected intravenously with Alexa-Fluor 488-BSA (10 mg/kg) and permeability was quantified as described in the methods section. Permeability was significantly increased in the diabetic uPAR+/+ or uPAR+/- mice compared with the non-diabetic controls (A). Permeability was not increased in the uPAR-/- mice. n=4-6. C, control; D, diabetic; *, *P*<0.01 *versus* non-diabetic control; #, *P*<0.01 *versus* uPAR+/+ and uPAR+/- diabetic. Gelatin zymograms prepared using vitreous samples collected from the same mice showed prominent increases in MMP9 activity in the uPAR+/+ and uPAR+/- diabetic mice. MMP9 activity was absent in retinas from uPAR-/- diabetic mice and non-diabetic controls (B). Retinal sections from diabetic and control mice were immunolabeled with anti-phospho-GSK3β antibody (green) and Texas red-isolectin B4 (red) (C). This analysis showed that phospho-GSK3β co-localizes with the lectin-positive vessels in the diabetic retina (arrows) and is present in microglial-like cells (arrowheads) in both control and diabetic retinas.

In order to directly demonstrate the role of uPAR in diabetes-induced breakdown of the blood-retinal barrier, diabetes was induced in uPAR-/- and littermate controls (uPAR+/+, uPAR+/-) by streptozotocin injection. Diabetes-induced BRB breakdown was quantified by analyzing extravasation of BSA-fluorescein. Vascular permeability was significantly increased (3-fold, p < 0.01) in diabetic congenic control mice as compared with the normoglycemic control mice (Figure 7A-B). The diabetic uPAR-/- mice showed no permeability increase over non-diabetic controls.

To further explore the role of the uPA/uPAR pathway in the diabetes-induced vascular permeability increase, gelatin zymography was used to determine the effects of uPAR deletion on MMP activities in vitreous collected from the uPAR-/- diabetic and non-diabetic mice and their littermate controls. This analysis showed prominent bands of MMP9 activity (gelatin lysis) in congenic control diabetic animals (Figure 7C). Strikingly, the diabetic uPAR-/- animals showed no detectable MMP9 band. In order to assess the specificity of the uPAR deletion in preventing MMP9 activation, we determined the effects of diabetes on MMP9 expression by quantitative PCR. This analysis showed significant increases in MMP9 mRNA in the retinas of all three genotypes of diabetic mice, suggesting that the protective effect of the gene deletion involves blockade of uPA-mediated proteolysis rather than modulating MMP-9 expression.

## Discussion

**Figure 8:**
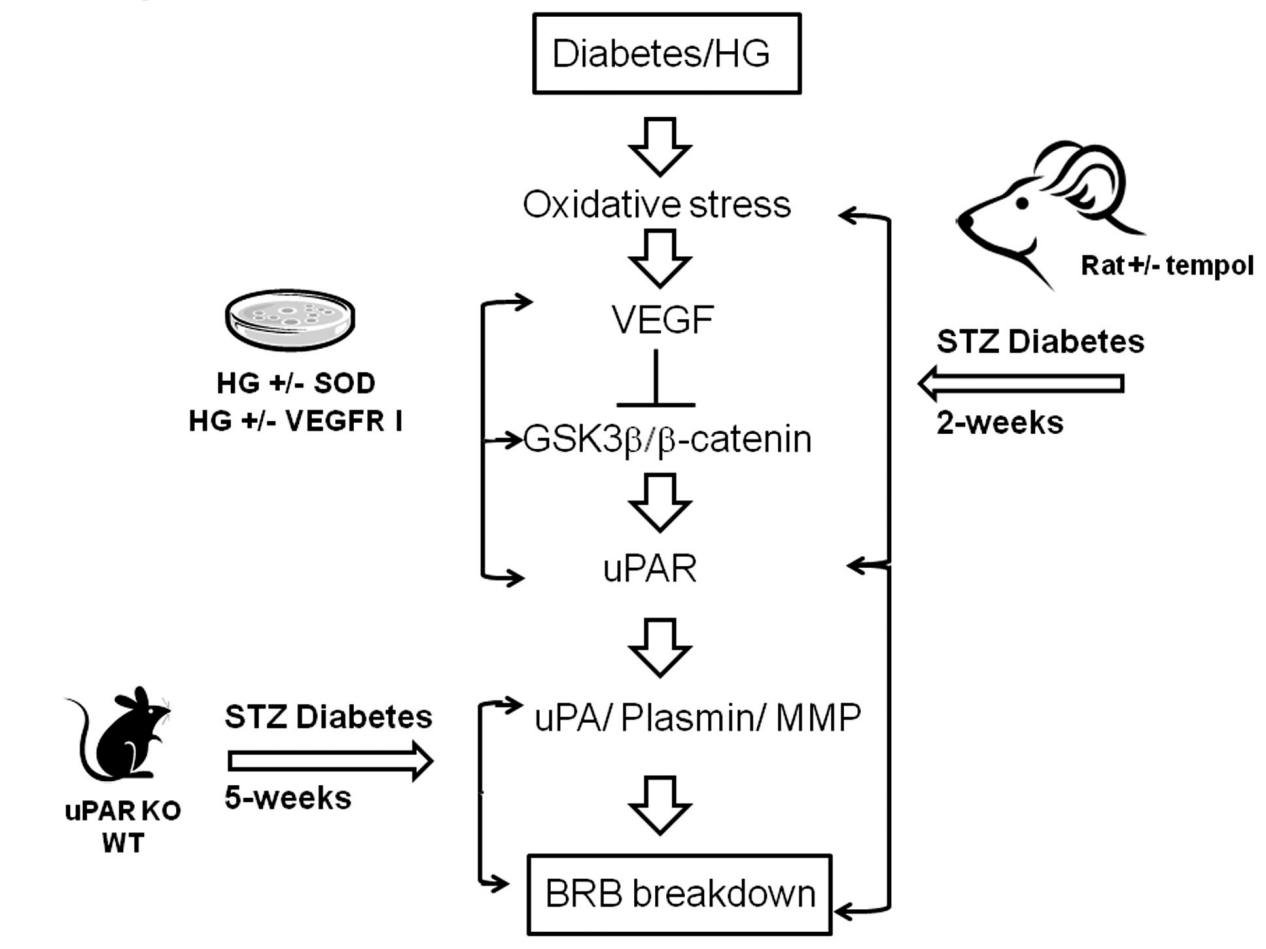
Hypothesized scheme of events for how diabetes/hyperglycemia induced oxidative stress triggers VEGF-mediated uPAR expression and consequent BRB breakdown, and the experimental approaches that produced the supporting data.

The main findings of this study are as follows: 1. Reducing superoxide anion blocked diabetes-induced vascular permeability in vivo and high glucose-induced expression of VEGF and uPAR *in vitro* (Figures 1, 2, 3). 2. The high glucose-induced increases in VEGF, uPAR and phosphorylation of GSK3β are mediated at least in part via autocrine activation of VEGFR (Figures 2, 3, 4). 3. High glucose and VEGF caused nuclear translocation of β-catenin and increased REC permeability in a uPA/uPAR dependent manner (Figures 5, 6). 4. Deletion of uPAR in mice preserved the BRB and blocked diabetes-induced increases in MMP9 activity (Figure 7). Based on these results we conclude that diabetes-induced oxidative stress triggers BRB breakdown by a mechanism that involves uPAR expression through VEGF-induced activation of the GSK-3 β/β-catenin signaling pathway (Figure 8).

Although diabetes and high glucose-induced increases in superoxide anion have been well-documented [10–12], the direct effects and possible mechanisms of reducing superoxide anion on BRB breakdown have not been fully elucidated. Here we show that treatment of diabetic rats with continuous infusion of the SOD mimetic tempol completely blocked diabetes-induced BRB breakdown. These protective effects were associated with significant decreases in lipid peroxidation and tyrosine nitration in the diabetic retina. These results are consistent with a recent report showing that administration of tempol prevented retinal tyrosine nitration and pericyte cell death and preserved BRB in Akita diabetic mice [31]. The latter study did not examine the direct effects of superoxide anion production within endothelial cells. However, overexpression of MnSOD in retinal endothelial cells has been shown to prevent diabetes-induced VEGF expression [12]. Therefore, we further explored the role of inhibiting superoxide anion formation in cultures of REC maintained in high glucose. Indeed, treatment with the cell permeable SOD blocked high glucose-induced increases in expression of VEGF, uPAR in REC. These observations in REC (Figures 2, 3) along with the finding that tempol significantly reduced diabetes-induced uPAR expression *in vivo* (Figure 1) indicate that high glucose induces VEGF and uPAR expression by increasing oxidative stress. These results lend further support to previous reports showing that diabetes-induced oxidative stress and peroxynitrite formation enhance VEGF expression [4,8], by a mechanism involved activation of the signal transducer and activator of transcription 3 [9]. The findings that HG-induced upregulation of VEGF and uPAR were partially blocked by the VEGFR1/2 inhibitor suggest an autocrine mechanism of VEGF/VEGFR activation. This result is consistent with previous reports that VEGF can induce its own expression in a variety of cell types, including vascular endothelial cells [32,33].

The role of VEGF in diabetic retinopathy and breakdown of the blood-retinal barrier is well established [34]. Diabetes/high glucose-induced increases in MMP9 activity in retinal tissues *in vivo* and retinal endothelial cells *in vitro* and alterations in composition of the perivascular extracellular matrix have been linked to retinal vascular injury and compromised barrier function [35–38]. Previous studies have also implicated increases in uPA and MMP expression and activities in breakdown of the blood retinal barrier [15,20,30,39,40]. Investigations using cultured retinal endothelial cells have shown that treatment with exogenous MMP9 and uPA causes an increase in paracellular permeability by a mechanism involving decreases in the tight junction protein occludin [14,26]. VEGF-induced migration and capillary morphogenesis has also been shown to be mediated by the induction uPAR gene expression [41]. Expression of uPAR is thought to provide a cellular-based proteolytic system that facilitates activation of uPA and plasminogen. Plasmin digests ECM components and activates pro-matrix metalloproteinases, including MMP9 (for review, please see 42). The localization of plasmin and metalloprotease proteolytic activities at the endothelial cell plasma membrane causes loosening of cell-cell and cell-matrix contacts which increases vascular permeability and releases serum proteins and ECM-bound growth factors. To our knowledge, actions of uPA and MMP9 have not been studied in non-vascular cells of the diabetic retina. However, the uPAR proteolytic system has been shown to be involved in retinal pigment epithelial cell migration [43] and has been linked to choroidal neovascularization in animal models of age related macular degeneration [44,45]. Activity of uPA and MMP9 has also been linked to gliosis and retinal ganglion cell death in experimental models of open angle glaucoma [46,47]).

We have shown previously that VEGF treatment induces GSK3β phosphorylation, nuclear translocation of β-catenin and increases uPAR gene expression in retinal endothelial cells [14,16,17]. The present study showed that high glucose treatment of retinal endothelial cells substantially increased the accumulation of β-catenin within both the cytosolic and nuclear compartments. Also, GSK3β was phosphorylated in a time-dependent manner following high glucose treatment. These observations indicate that high glucose causes GSK3β phosphorylation and deactivation, leading to stabilization and nuclear translocation of β-catenin. Our studies in mice confirmed the involvement of the uPA/uPAR system in diabetes-induced breakdown of the blood-retinal barrier.

Although anti-VEGF therapies have proven successful in reducing vascular leakage and macular edema in diabetic patients [48], most patients require repeated intraocular injections and some do not respond. In addition, depriving the diabetic retina of VEGF can exacerbate diabetes-induced neurodegeneration due to lack of neurotrophic support to neurons. Our studies identified several molecular targets downstream from VEGF that can be further developed as anti-permeability therapies. In summary, as depicted in the graphical abstract (Figure 8), we have used animal and tissue culture models to demonstrate that diabetes-induced oxidative stress triggers BRB breakdown by a mechanism that involves uPAR expression through VEGF-induced phosphorylation and deactivation of the GSK-3 β/β-catenin signaling pathway. Furthermore, we show that in the presence of VEGFR2 inhibitor, the glucose-induced uPAR expression is abrogated (Figure 3B). This indicates that high glucose utilizes the VEGF/VEGFR2 signaling pathway to regulate β-catenin transcriptional activity and uPAR gene expression. Our data also indicate that hyperglycemia induced upregulation of VEGF and activation of VEGFR2 have a role in increasing uPAR expression.

## References

[1] Cunha-Vaz JG (1978) Pathophysiology of diabetic retinopathy. Br J Ophthalmol 62: 351-355. doi:10.1136/bjo.62.6.351. PubMed: 666982. 666982 10.1136/bjo.62.6.351 PMC1043233

[2] Gardner TW, Antonetti DA, Barber AJ, LaNoue KF, Levison SW (2002) Diabetic retinopathy: more than meets the eye. Surv Ophthalmol 47 Suppl 2: S253-S262. doi:10.1016/S0039-6257(02)00387-9. PubMed: 12507627. 12507627 10.1016/s0039-6257(02)00387-9

[3] Tilton RG (2002) Diabetic vascular dysfunction: links to glucose-induced reductive stress and VEGF. Microsc Res Tech 57: 390-407. doi:10.1002/jemt.10092. PubMed: 12112445. 12112445 10.1002/jemt.10092

[4] El-Remessy AB, Behzadian MA, Abou-Mohamed G, Franklin T, Caldwell RW et al. (2003) Experimental diabetes causes breakdown of the blood-retina barrier by a mechanism involving tyrosine nitration and increases in expression of vascular endothelial growth factor and urokinase plasminogen activator receptor. Am J Pathol 162: 1995-2004. doi:10.1016/S0002-9440(10)64332-5. PubMed: 12759255. 12759255 10.1016/S0002-9440(10)64332-5 PMC1868147

[5] Malecaze F, Clamens S, Simorre-Pinatel V, Mathis A, Chollet P et al. (1994) Detection of vascular endothelial growth factor messenger RNA and vascular endothelial growth factor-like activity in proliferative diabetic retinopathy. Arch Ophthalmol 112: 1476-1482. doi:10.1001/archopht.1994.01090230090028. PubMed: 7980139. 7980139 10.1001/archopht.1994.01090230090028

[6] Murata T, Ishibashi T, Khalil A, Hata Y, Yoshikawa H et al. (1995) Vascular endothelial growth factor plays a role in hyperpermeability of diabetic retinal vessels. Ophthal Res 27: 48-52. doi:10.1159/000267567. PubMed: 7596559. 10.1159/000267567 7596559

[7] Caldwell RB, Bartoli M, Behzadian MA, El-Remessy AE, Al-Shabrawey M et al. (2005) Vascular endothelial growth factor and diabetic retinopathy: role of oxidative stress. Curr Drug Targets 6: 511-524. doi:10.2174/1389450054021981. PubMed: 16026270. 16026270 10.2174/1389450054021981

[8] El-Remessy AB, Al-Shabrawey M, Khalifa Y, Tsai NT, Caldwell RB et al. (2006) Neuroprotective and blood-retinal barrier-preserving effects of cannabidiol in experimental diabetes. Am J Pathol 168: 235-244. doi:10.2353/ajpath.2006.050500. PubMed: 16400026. 16400026 10.2353/ajpath.2006.050500 PMC1592672

[9] Platt DH, Bartoli M, El-Remessy AB, Al-Shabrawey M, Lemtalsi T et al. (2005) Peroxynitrite increases VEGF expression in vascular endothelial cells via STAT3. Free Radic Biol Med 39: 1353-1361. doi:10.1016/j.freeradbiomed.2005.06.015. PubMed: 16257644. 16257644 10.1016/j.freeradbiomed.2005.06.015

[10] El-Remessy AB, Abou-Mohamed G, Caldwell RW, Caldwell RB (2003) High glucose-induced tyrosine nitration in endothelial cells: role of eNOS uncoupling and aldose reductase activation. Invest Ophthalmol Vis Sci 44: 3135-3143. doi:10.1167/iovs.02-1022. PubMed: 12824263. 12824263 10.1167/iovs.02-1022

[11] Kowluru RA, Kowluru V, Xiong Y, Ho YS (2006) Overexpression of mitochondrial superoxide dismutase in mice protects the retina from diabetes-induced oxidative stress. Free Radic Biol Med 41: 1191-1196. doi:10.1016/j.freeradbiomed.2006.01.012. PubMed: 17015165. 17015165 10.1016/j.freeradbiomed.2006.01.012

[12] Goto H, Nishikawa T, Sonoda K, Kondo T, Kukidome D et al. (2008) Endothelial MnSOD overexpression prevents retinal VEGF expression in diabetic mice. Biochem Biophys Res Commun 366: 814-820. doi:10.1016/j.bbrc.2007.12.041. PubMed: 18083119. 18083119 10.1016/j.bbrc.2007.12.041

[13] Ali TK, El-Remessy AB (2009) Diabetic retinopathy: current management and experimental therapeutic targets. Pharmacotherapy 29: 182-192. doi:10.1592/phco.29.2.182. PubMed: 19170588. 19170588 10.1592/phco.29.2.182

[14] Behzadian MA, Windsor LJ, Ghaly N, Liou G, Tsai NT et al. (2003) VEGF-induced paracellular permeability in cultured endothelial cells involves urokinase and its receptor. FASEB J 17: 752-754. PubMed: 12594181. 12594181 10.1096/fj.02-0484fje

[15] Navaratna D, Menicucci G, Maestas J, Srinivasan R, McGuire P et al. (2008) A peptide inhibitor of the urokinase/urokinase receptor system inhibits alteration of the blood-retinal barrier in diabetes. FASEB J 22: 3310-3317. doi:10.1096/fj.08-110155. PubMed: 18559877. 18559877 10.1096/fj.08-110155 PMC2518254

[16] Yang J, Caldwell RB, Behzadian MA (2012) Blockade of VEGF-induced GSK/beta-catenin signaling, uPAR expression and increased permeability by dominant negative p38alpha. Exp Eye Res 100: 101-108. doi:10.1016/j.exer.2012.03.011. PubMed: 22564969. 22564969 10.1016/j.exer.2012.03.011 PMC3798053

[17] Yang J, Duh EJ, Caldwell RB, Behzadian MA (2010) Antipermeability function of PEDF involves blockade of the MAP kinase/GSK/beta-catenin signaling pathway and uPAR expression. Invest Ophthalmol Vis Sci 51: 3273-3280. doi:10.1167/iovs.08-2878. PubMed: 20089873. 20089873 10.1167/iovs.08-2878 PMC2891479

[18] Pepper MS (2001) Role of the matrix metalloproteinase and plasminogen activator-plasmin systems in angiogenesis. Arterioscler Thromb Vasc Biol 21: 1104-1117. doi:10.1161/hq0701.093685. PubMed: 11451738. 11451738 10.1161/hq0701.093685

[19] Ellis V, Behrendt N, Danø K (1991) Plasminogen activation by receptor-bound urokinase. A kinetic study with both cell-associated and isolated receptor. J Biol Chem 266: 12752-12758. PubMed: 1829461. 1829461

[20] Giebel SJ, Menicucci G, McGuire PG, Das A (2005) Matrix metalloproteinases in early diabetic retinopathy and their role in alteration of the blood-retinal barrier. Lab Invest 85: 597-607. doi:10.1038/labinvest.3700251. PubMed: 15711567. 15711567 10.1038/labinvest.3700251

[21] Dejana E, Corada M, Lampugnani MG (1995) Endothelial cell-to-cell junctions. FASEB J 9: 910-918. PubMed: 7615160. 7615160

[22] Lampugnani MG, Corada M, Caveda L, Breviario F, Ayalon O et al. (1995) The molecular organization of endothelial cell to cell junctions: differential association of plakoglobin, beta-catenin, and alpha- catenin with vascular endothelial cadherin (VE-cadherin). J Cell Biol 129: 203-217. doi:10.1083/jcb.129.1.203. PubMed: 7698986. 7698986 10.1083/jcb.129.1.203 PMC2120375

[23] Sadot E, Simcha I, Iwai K, Ciechanover A, Geiger B et al. (2000) Differential interaction of plakoglobin and beta-catenin with the ubiquitin-proteasome system. Oncogene 19: 1992-2001. doi:10.1038/sj.onc.1203519. PubMed: 10803460. 10803460 10.1038/sj.onc.1203519

[24] Aberle H, Bauer A, Stappert J, Kispert A, Kemler R (1997) beta-catenin is a target for the ubiquitin-proteasome pathway. EMBO J 16: 3797-3804. doi:10.1093/emboj/16.13.3797. PubMed: 9233789. 9233789 10.1093/emboj/16.13.3797 PMC1170003

[25] Mann B, Gelos M, Siedow A, Hanski ML, Gratchev A et al. (1999) Target genes of beta-catenin-T cell-factor/lymphoid-enhancer-factor signaling in human colorectal carcinomas. Proc Natl Acad Sci U S A 96: 1603-1608. doi:10.1073/pnas.96.4.1603. PubMed: 9990071. 9990071 10.1073/pnas.96.4.1603 PMC15532

[26] Behzadian MA, Wang XL, Windsor LJ, Ghaly N, Caldwell RB (2001) TGF-beta increases retinal endothelial cell permeability by increasing MMP-9: possible role of glial cells in endothelial barrier function. Invest Ophthalmol Vis Sci 42: 853-859. PubMed: 11222550. 11222550

[27] Brands MW, Bell TD, Gibson B (2004) Nitric oxide may prevent hypertension early in diabetes by counteracting renal actions of superoxide. Hypertension 43: 57-63. PubMed: 14656952. 14656952 10.1161/01.HYP.0000104524.25807.EE

[28] Al-Shabrawey M, Rojas M, Sanders T, Behzadian A, El-Remessy A et al. (2008) Role of NADPH oxidase in retinal vascular inflammation. Invest Ophthalmol Vis Sci 49: 3239-3244. doi:10.1167/iovs.08-1755. PubMed: 18378574. 18378574 10.1167/iovs.08-1755 PMC3798055

[29] Mohamed IN, Soliman SA, Alhusban A, Matragoon S, Pillai BA et al. (2012) Diabetes exacerbates retinal oxidative stress, inflammation, and microvascular degeneration in spontaneously hypertensive rats. Mol Vis 18: 1457-1466. PubMed: 22736937. 22736937 PMC3380918

[30] Navaratna D, McGuire PG, Menicucci G, Das A (2007) Proteolytic degradation of VE-cadherin alters the blood-retinal barrier in diabetes. Diabetes 56: 2380-2387. doi:10.2337/db06-1694. PubMed: 17536065. 17536065 10.2337/db06-1694

[31] Zou MH, Li H, He C, Lin M, Lyons TJ et al. (2011) Tyrosine nitration of prostacyclin synthase is associated with enhanced retinal cell apoptosis in diabetes. Am J Pathol 179: 2835-2844. doi:10.1016/j.ajpath.2011.08.041. PubMed: 22015457. 22015457 10.1016/j.ajpath.2011.08.041 PMC3260825

[32] Byrne AM, Bouchier-Hayes DJ, Harmey JH (2005) Angiogenic and cell survival functions of vascular endothelial growth factor (VEGF). J Cell Mol Med 9: 777-794. doi:10.1111/j.1582-4934.2005.tb00379.x. PubMed: 16364190. 16364190 10.1111/j.1582-4934.2005.tb00379.x PMC6740098

[33] Bartoli M, Platt D, Lemtalsi T, Gu X, Brooks SE et al. (2003) VEGF differentially activates. Stat 3 in microvascular endothelial cells. Faseb J 17: 1562-1564 10.1096/fj.02-1084fje 12824281

[34] Penn JS, Madan A, Caldwell RB, Bartoli M, Caldwell RW et al. (2008) Vascular endothelial growth factor in eye disease. Prog Retin Eye Res 27: 331-371. doi:10.1016/j.preteyeres.2008.05.001. PubMed: 18653375. 18653375 10.1016/j.preteyeres.2008.05.001 PMC3682685

[35] Kowluru RA (2010) Role of matrix metalloproteinase-9 in the development of diabetic retinopathy and its regulation by H-Ras. Invest Ophthalmol Vis Sci 51: 4320-4326. doi:10.1167/iovs.09-4851. PubMed: 20220057. 20220057 10.1167/iovs.09-4851 PMC2910650

[36] Kowluru RA, Mohammad G, Dos Santos JM, Zhong Q (2011) Abrogation of MMP-9 Gene Protects Against the Development of Retinopathy in Diabetic Mice by Preventing Mitochondrial Damage. Diabetes, 60: 3023–33. PubMed: 21933988. 21933988 10.2337/db11-0816 PMC3198054

[37] Chronopoulos A, Tang A, Beglova E, Trackman PC, Roy S (2010) High glucose increases lysyl oxidase expression and activity in retinal endothelial cells: mechanism for compromised extracellular matrix barrier function. Diabetes 59: 3159-3166. doi:10.2337/db10-0365. PubMed: 20823103. 20823103 10.2337/db10-0365 PMC2992778

[38] Roy S, Ha J, Trudeau K, Beglova E (2010) Vascular basement membrane thickening in diabetic retinopathy. Curr Eye Res 35: 1045-1056. doi:10.3109/02713683.2010.514659. PubMed: 20929292. 20929292 10.3109/02713683.2010.514659

[39] Das A, McGuire PG, Eriqat C, Ober RR, DeJuan E Jr. et al. (1999) Human diabetic neovascular membranes contain high levels of urokinase and metalloproteinase enzymes. Invest Ophthalmol Vis Sci 40: 809-813. PubMed: 10067990. 10067990

[40] Noda K, Ishida S, Inoue M, Obata K, Oguchi Y et al. (2003) Production and activation of matrix metalloproteinase-2 in proliferative diabetic retinopathy. Invest Ophthalmol Vis Sci 44: 2163-2170. doi:10.1167/iovs.02-0662. PubMed: 12714657. 12714657 10.1167/iovs.02-0662

[41] Prager GW, Breuss JM, Steurer S, Mihaly J, Binder BR (2004) Vascular endothelial growth factor (VEGF) induces rapid prourokinase (pro-uPA) activation on the surface of endothelial cells. Blood 103: 955-962. PubMed: 14525763. 14525763 10.1182/blood-2003-07-2214

[42] Mondino A, Blasi F (2004) uPA and uPAR in fibrinolysis, immunity and pathology. Trends Immunol 25: 450-455. doi:10.1016/j.it.2004.06.004. PubMed: 15275645. 15275645 10.1016/j.it.2004.06.004

[43] Elner SG, Elner VM, Kindzelskii AL, Horino K, Davis HR et al. (2003) Human RPE cell lysis of extracellular matrix: functional urokinase plasminogen activator receptor (uPAR), collagenase and elastase. Exp Eye Res 76: 585-595. doi:10.1016/S0014-4835(03)00028-9. PubMed: 12697422. 12697422 10.1016/s0014-4835(03)00028-9

[44] Das A, Boyd N, Jones TR, Talarico N, McGuire PG (2004) Inhibition of choroidal neovascularization by a peptide inhibitor of the urokinase plasminogen activator and receptor system in a mouse model. Arch Ophthalmol 122: 1844-1849. doi:10.1001/archopht.122.12.1844. PubMed: 15596589. 15596589 10.1001/archopht.122.12.1844

[45] Rakic JM, Lambert V, Munaut C, Bajou K, Peyrollier K et al. (2003) Mice without uPA, tPA, or plasminogen genes are resistant to experimental choroidal neovascularization. Invest Ophthalmol Vis Sci 44: 1732-1739. doi:10.1167/iovs.02-0809. PubMed: 12657615. 12657615 10.1167/iovs.02-0809

[46] Ganesh BS, Chintala SK (2011) Inhibition of reactive gliosis attenuates excitotoxicity-mediated death of retinal ganglion cells. PLOS ONE 6: e18305. doi:10.1371/journal.pone.0018305. PubMed: 21483783. 21483783 10.1371/journal.pone.0018305 PMC3069086

[47] Chintala SK (2006) The emerging role of proteases in retinal ganglion cell death. Exp Eye Res 82: 5-12. doi:10.1016/j.exer.2005.07.013. PubMed: 16185688. 16185688 10.1016/j.exer.2005.07.013

[48] Brown DM, Nguyen QD, Marcus DM, Boyer DS, Patel S et al. (2013) Long-term Outcomes of Ranibizumab Therapy for Diabetic Macular Edema: The 36-Month Results from Two Phase III Trials: RISE and RIDE. Ophthalmology. PubMed: 23706949. 10.1016/j.ophtha.2013.02.034 23706949

