# Correction: Diabetes-Induced Superoxide Anion and Breakdown of the Blood-Retinal Barrier: Role of the VEGF/uPAR Pathway

**DOI:** 10.1371/journal.pone.0186749

**Published:** 2017-10-16

**Authors:** Azza B. El-Remessy, Telina Franklin, Nagla Ghaley, Jinling Yang, Michael W. Brands, Ruth B. Caldwell, Mohamed Ali Behzadian

After the publication of the article, concerns were raised about Fig 4A in the article, as follows:

In panel P-GSK-3, the NG lane and the VEGF + VEGFRI lane are duplicated.

In panel GSK-3, the VEGF and VEGF+VEGFRI lanes duplicate the HG-1d and HG-3d lanes.

The authors have acknowledged errors in the preparation of these panels. Following evaluation of the data provided by the authors and an investigation by the University of Georgia, we are issuing a correction to correct Fig 4. The corrected figure and raw data for the results are included in this Correction.

The authors apologize for the errors in the original figure.

**Fig 4:**
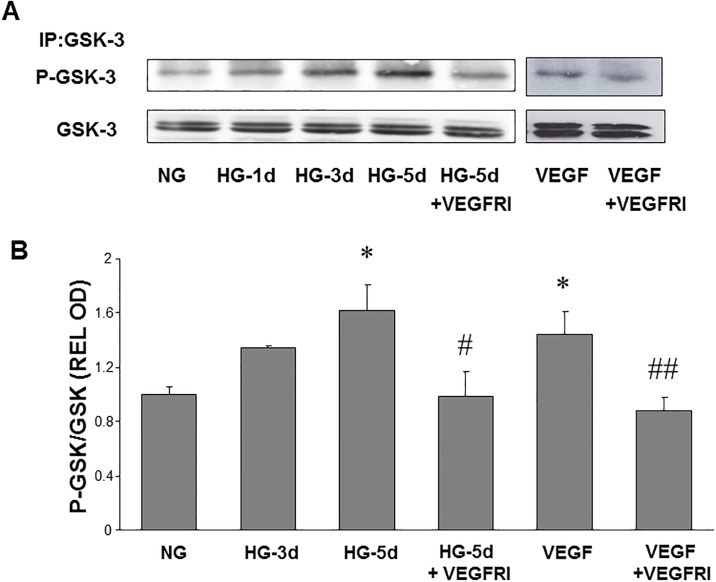
High glucose-induced phosphorylation of GSK3β. Endothelial cells were grown in serum-free medium containing normal glucose (NG, 5.5 mM) or high glucose (HG, 25 mM) with or without VEGFR inhibitor (VEGFRI) for 1 to 5 days. Equal amounts of SDS extracted protein samples were immunoprecipitated (IP) by anti-GSK3β antibody and subjected to SDS-PAGE and Western blotting (A). Densitometric analysis of phospho-GSK3β bands, normalized for the corresponding GSK3β bands, showed that phosho-GSK3β levels were significantly increased by HG or VEGF treatment and that this effect was reduced in VEGFI treated samples compared to HG or VEGF treated endothelial cells (B). * = P<0.05 vs NG, # = P<0.05 vs HG5, # # = P<0.05 vs VEGF.

## Supporting information

S1 Dataset Supplementary data. This file contains the raw data underlying the corrected Fig 4.
(TIF)
