# Supplementary figures and images for: Correction: Diabetes-Induced Superoxide Anion and Breakdown of the Blood-Retinal Barrier: Role of the VEGF/uPAR Pathway
Source: PLoS One. 2017 Oct 16;12(10):e0186749. doi: 10.1371/journal.pone.0186749 (PMC5643131; doi:10.1371/journal.pone.0186749)

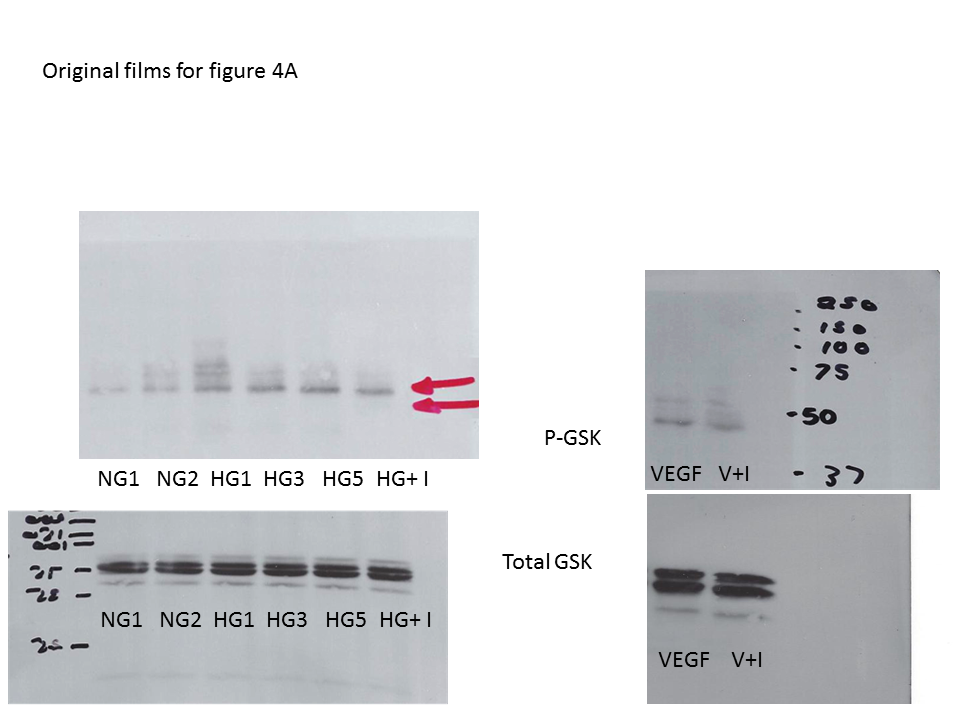

Supplement: S1 Dataset — This file contains the raw data underlying the corrected Fig 4. (TIF) [file pone.0186749.s001.tif]
